# Nursing personnel and speech-language therapists communication practices in dysphagia management

**DOI:** 10.4102/sajcd.v72i2.1129

**Published:** 2025-11-23

**Authors:** Alida de Beer, Athenkosi Mpiti, Zeenat Ganie, Lesedi Rakgase, Sinovuyo Mduzulwana, Aldon Barend

**Affiliations:** 1 Division of Speech-Language and Hearing Therapy, Faculty of Medicine and Health Sciences, Stellenbosch University, Cape Town, South Africa

**Keywords:** dysphagia, communication, interdisciplinary, speech-language therapists, nursing personnel

## Abstract

**Background:**

Communication between speech-language therapists (SLTs) and nursing personnel is essential for safe dysphagia management, as unclear exchanges can compromise patient outcomes and care continuity. In South Africa, high patient loads, limited dysphagia training, and linguistic diversity create additional challenges, making effective interprofessional communication crucial.

**Objectives:**

This study aimed to identify communication practices related to dysphagia in exchanges between SLTs and nursing personnel, focusing on mutual understanding, openness in interactions and frustration experienced during communication. It also explored nursing personnel’s preferred communication modes when discussing feeding and swallowing management.

**Method:**

A quantitative, cross-sectional design was used. Data were collected from 18 nurses by means of an adapted Nurse-Physician Communication Scale. The survey was distributed electronically across South Africa via multiple channels. Purposive sampling targeted nursing personnel with at least 6 months’ experience in managing patients with dysphagia.

**Results:**

Findings showed that while some mutual understanding exists, nursing personnel often felt misunderstood during interactions with SLTs. Reduced mutual understanding and openness correlated with dissatisfaction in communication. Nursing personnel frequently perceived much of the dysphagia-related information as irrelevant, which increased frustration. Nurses expressed a preference for face-to-face verbal and written communication over electronic modes.

**Conclusion:**

The study highlights the need to improve communication practices employed in dysphagia management to support successful interactions and optimal patient management. Strengthening interdisciplinary communication between SLTs and nursing personnel can enhance patient outcomes, increase nursing satisfaction, and promote better teamwork.

## Introduction

In the rapidly evolving contemporary medical environment, effective communication between healthcare professionals is essential. It supports timely and efficient operations, fosters connections between professionals and other caregivers, and ultimately ensures that patients receive the best, most immediate care possible (Gleeson et al., 2023). Communication in healthcare is widely recognised as a fundamental clinical skill (Ferreira-Padilla et al., 2015). It not only helps establish strong working relationships among healthcare providers but also paves the way for accurate diagnosis and effective treatment. For communication to be successful in healthcare settings, professionals must be able to convey complex or technical information clearly so that everyone involved, regardless of background, understands the patient’s health status and care needs.

This clarity becomes especially crucial when managing feeding and swallowing difficulties, also known as dysphagia, where the two-way exchange of information is vital. Two-way communication, or bidirectional exchange, is critical in healthcare teams because it ensures that information is not only transmitted but also accurately received, interpreted and applied. In dysphagia management, this means that speech-language therapists (SLTs) must clearly convey recommendations regarding patient positioning, swallowing manoeuvres and diet modifications, while nurses provide real-time feedback on patient responses, challenges in implementation and any adverse events observed during feeding. Without this reciprocal flow of information, critical details may be misunderstood, leading to inconsistent care, increased risk of aspiration, dehydration or malnutrition, and overall diminished patient safety (Barnard et al., 2022; Stühlinger et al., 2019). In acute and rehabilitation care settings, interaction between SLTs and nursing personnel forms part of an interdisciplinary approach to dysphagia management. According to Barnard et al. (2022), the communication and interaction mechanisms through which various dysphagia risk factors are shared between SLTs and nursing personnel are poorly understood.

Speech-language therapists focus on ensuring safe feeding practices while preventing secondary complications such as dehydration, aspiration pneumonia and malnutrition (Clark & Ebersole, 2018; Hines et al., 2014; Vose et al., 2014). Safe feeding can be achieved by recommending individualised dysphagia management strategies, including diet modifications, specific swallowing techniques and manoeuvres, and appropriate patient positioning (O’Keeffe, 2018). Furthermore, SLTs strive to maintain patients’ quality of life by incorporating individual preferences and family beliefs into care plans, and by adapting these plans as patient needs evolve (Shee et al., 2019).

Nursing professionals play an equally vital role in managing feeding and swallowing difficulties as they need to make the feeding and swallowing decisions in the moment, while SLTs are available intermittently (Barnard et al., 2022). Their main responsibility is to implement the management strategies recommended by SLTs and dieticians, and to monitor the patient for risk of aspiration. Their tasks include identifying aspiration risks, referring patients to SLTs for further assessment and following through with interventions. According to Seedat and Strime (2021), nurses perceive their involvement in dysphagia management as encompassing five key areas: ensuring patients receive the correct diet, making timely referrals, maintaining oral hygiene, administering medication and participating in training. In addition to these tasks, nurses are regularly involved in directly assisting patients during feeding, performing prescribed swallowing techniques and manoeuvres, and monitoring patient responses to interventions (Hines et al., 2014; Pierpoint & Pillay, 2020). They provide essential frontline care, support patients emotionally and physically, educate family members and help ensure that care is both consistent and holistic. Their constant presence positions them as patient advocates, coordinators of care and key mediators in the interdisciplinary healthcare team (Bloom et al., 2022).

Communication practices employed in exchanges between SLTs and nursing personnel, however, can vary significantly across different settings and countries. In South Africa, communication between SLTs and nursing personnel is influenced by several contextual and systemic challenges. The country’s multilingual landscape, where English and Afrikaans dominate while many indigenous languages are marginalised, can lead to misunderstandings and impede clear information exchange. Informal interpreting practices, such as using family members or untrained staff as translators, often compromise accuracy and confidentiality (Taylor & Kazembe, 2024). Interprofessional collaboration is further limited by hierarchical structures, a lack of shared training and insufficient opportunities for joint decision-making, a constraint, which can result in fragmented care and reduced patient safety (Mohamed et al., 2024). Nursing staff also face multiple barriers to implementing SLT recommendations for dysphagia management, including limited training, unfamiliar terminology, high workloads and time constraints. Cultural differences and contextual factors may also contribute to misinterpretations of patient behaviour and care instructions.

It is evident that these conditions, which may lead to ineffective communication between SLTs and nurses, could compromise the quality of patient care (Barnard et al., 2022; Pillay, 2009; Robbertse & De Beer, 2020). As pointed out by Tanner and Culbertsome (2014), poor feeding and swallowing management often result from such communication breakdowns. One significant issue in South Africa is the shortage of SLTs. With an estimated SLT-to-patient ratio of 1:25 000, therapists are overwhelmed by the demand, which hinders their ability to collaborate effectively with nursing staff (Kathard & Pillay, 2013). This shortage inevitably further impacts the frequency and quality of communication and teamwork between SLTs and nursing personnel.

Effective communication in nursing is fundamentally linked to cohesive teamwork and trusting relationships; nurses who are empowered through collaboration and strong communication skills are better able to deliver patient-centred care and respond adaptively to clinical challenges (Dlamini & Park, 2024). Effective communication in collaborative healthcare involves a genuine exchange of patient care information, the cognitive capacity to process and apply this information, and the opportunity to share meaningful communication spaces (Barnard et al., 2020). Furthermore, strong interprofessional relationships are crucial for enhancing patient outcomes, encouraging collaborative practice and fostering mutual respect (Seedat & Strime, 2021). It is unfortunate, therefore, that communication between SLTs and nursing staff is often lacking. A literature review by Barnard et al. (2020) found that SLTs frequently observed that nursing personnel were not following the recommended safety advice for dysphagia patients. Miscommunication or loss of key information during discussions about feeding and swallowing management can significantly compromise care. According to Barlow et al. (2023), effective communication requires that a message be accurately encoded, transmitted, decoded and understood. For clinical communication to be effective, it must be comprehensive, clear, brief and timely (Bramhall, 2014; Wang et al., 2018). After investigating the impact on patient care of the quality of communication between nurses and physicians, Schmid and Svarstad (2002) outlined four dimensions that determine the effectiveness of communication in healthcare settings: mutual understanding, openness, frustration during interactions and overall satisfaction with communication exchanges. These dimensions warrant further scrutiny.

Mutual understanding in healthcare teams, fostered by the use of a shared professional language, is fundamental not only to enhancing care quality but also to improving job satisfaction, through mechanisms such as relational coordination and psychological safety (Stühlinger et al., 2019). Mutual understanding involves clearly interpreting verbal and non-verbal instructions, seeking clarification when needed, sharing opinions and actively checking each other’s observations and assessments. Such open dialogue helps ensure that care decisions are based on comprehensive and shared insights.

Openness in communication refers to honesty and transparency during the exchange of thoughts, ideas and concerns. It is fostered through active listening, honesty, trust and supportive interactions (Schiller & Cui, 2010). Openness is intrinsically linked to mutual understanding, and together these two aspects improve communication practices and ultimately patient care. Furthermore, open communication contributes significantly to individual performance and team functioning in clinical settings (Shrivastava & Prasad, 2019).

Contrarily, poor communication between healthcare professionals can lead to frustration and ultimately have serious consequences. It often results in miscommunication, delays in patient management and fragmented documentation, especially in acute care settings. Such issues compromise timely and accurate management implementation, potentially prolonging hospital stays and negatively affecting patient satisfaction (Alder, 2025). Emotional responses such as frustration often arise when communication breaks down, further straining team dynamics.

Studies conducted by Barnard et al. (2020) and Dondorf et al. (2015) have shown that current communication practices in exchanges between SLTs and nursing staff are often ineffective. In this context, ‘ineffective’ refers to limited mutual understanding and reduced communication openness, which lead to poor implementation of the SLT’s recommendations. A major barrier is the use of unfamiliar or obscure terminology, which can cause uncertainty among the nursing personnel. A second barrier involves the impact of technology. The shift towards digital communication technologies, while increasing efficiency, has reduced face-to-face interaction, which may lead to message ambiguity, especially in complex clinical situations. These technologies influence communication practices through factors such as the richness of the media utilised and the location or availability of devices. When healthcare professionals are dispersed and dependent on technology, rather than working side-by-side, the chances of misinterpreting messages increase (Manojlovich et al., 2015). The third barrier – aspects such as shortage of staff, time constraints and limited resources – influences both implementation of recommendations for dysphagia management and communication practices (Barnard et al., 2022)

Ineffective communication can jeopardise patient safety and escalate healthcare costs (Wang et al., 2018). Despite these challenges, both SLTs and nurses continue to fulfil their essential roles in the interdisciplinary management of dysphagia, aiming to improve safety and patient outcomes. Evaluating current communication practices between SLTs and nursing personnel should clearly be accorded high priority. Effective communication underpins nurses’ contributions to prevention, treatment, rehabilitation, education and health promotion in dysphagia care (Kourkouta & Papathanasiou, 2014). Therefore, the purpose of this research is to determine the current communication practices in exchanges between nursing personnel and SLTs in the context of caring for patients with feeding and swallowing difficulties. Specifically, the study sought to determine the degree of mutual understanding between nursing personnel and SLTs within the multidisciplinary team, as well as to investigate the openness of their interactions. In addition, the research aimed to examine the appropriateness and adequacy of communication patterns, while also identifying any frustrations experienced during these exchanges. Finally, the study set out to identify the preferred modes of communication selected by nursing personnel and SLTs when engaging in dysphagia management interactions.

## Research methods and design

### Design

A cross-sectional descriptive, quantitative design was used to explore the current communication practices in exchanges between nursing personnel and SLTs in the management of patients with dysphagia.

### Setting

Participants in this study include nursing personnel who are currently employed in South Africa and provide patient care for individuals with feeding and/or swallowing disorders. Nurses from both the private and public sectors were included. According to the 2030 Human Resources for Health Strategy: Investing in the Health Workforce for Universal Health Coverage (2020), there are 71 707 professional nurses, 31 039 enrolled nurses, and 33 821 nursing assistants in South Africa. In both the public and private sectors, there is currently one nurse (registered, enrolled, or auxiliary) for every 213 patients. They work in hospitals, clinics, government departments, the South African Defence Force, health and welfare organisations, municipalities, industrial organisations, nursing agencies, self-employment as private nurses, schools and outpatient care facilities (South African Nursing Council, 2019).

### Study population and sampling strategy

The study included nurses caring for patients experiencing feeding and/or swallowing difficulties in either private or public healthcare settings within South Africa. Participants had to have at least 6 months of experience working with patients with feeding and/or swallowing difficulties to ensure the reliability and validity of responses. Eligibility was confirmed through the completion of a survey.

A non-probability convenience sampling method was deemed appropriate because of the descriptive and explorative nature of the research study. This sampling method allowed researchers to access readily available participants who met the inclusion criteria and had experience with the phenomena that were investigated. While convenience sampling has limitations regarding generalisability, it was suitable for this study as it facilitated the collection of contextually relevant data from a specialised clinical population that can otherwise be difficult to access because of staff shortages and time constraints.

Because of the descriptive nature of the research, no set sample size was required. Data were collected via targeted professional networks to increase the reach of the research study and to ensure inclusion of participants from a variety of clinical contexts. Recruitment strategies included posting invitations in Facebook nursing groups, circulating the survey via hospital administration channels and using personal contacts within professional networks. This multi-pronged recruitment process ensured that both public and private sector nursing practitioners were represented in the sample, thereby enriching the descriptive findings of the study. Despite the multiple recruitment strategies employed, a limited number of participants were recruited. Reasons for the small number of participants include the short recruitment period of 3 weeks, the specificity of inclusion criteria and the workload of nursing personnel. Because of the explorative and descriptive nature of the investigation, the small sample size was deemed adequate to achieve the objectives of the study.

### Data collection

Data were collected using an e-survey that was based on Schmid and Svarstad’s (2002) Nurse-Physician Communication Scale. The Nurse-Physician Communication Scale rates the quality of communication between nurses and physicians. The scale had 18 items and 4 dimensions: communication openness, mutual understanding, frustration with interaction, relevance and communication satisfaction (Schmid & Svarstad, 2002). Although the Nurse-Physician Communication Scale provided a valuable framework, the communication interaction and difficulties found in dysphagia management might differ from general communication practices in exchanges between nursing personnel and physicians, because of the urgency of care and interdisciplinary reliance on feedback. Therefore, the scale was modified and adjusted to ensure relevance and accuracy for this study. Modifications included replacing *the physician* with *SLTs*. In addition, new items were added to address aspects of preferred modes of communication, resulting in a 32-item e-survey. The scale now focuses on communication quality as well as preferred communication methods to highlight the interdisciplinary care in dysphagia management. The modified survey was sent to an expert panel (SLTs working in dysphagia) for comment regarding comprehensibility, fluency and the appropriateness and relevance of questions used in the survey (Leon et al., 2011). Feedback from the expert panel was mostly editorial. The necessary changes were implemented before the final survey was distributed to nursing personnel.

### Data analysis

Data were obtained via a survey completed by nursing personnel using a mandatory 5-point Likert scale. An Excel spreadsheet containing all the data responses was created from Google Forms. Responses to each item were also converted into percentages to facilitate interpretation and comparison across items.

The use of descriptive statistics was particularly suited to this study, as the aim was exploratory and focused on mapping communication practices rather than testing hypotheses. Presenting results in both frequency counts and percentages ensured that findings were accessible and could be meaningfully interpreted within the context of interprofessional collaboration in dysphagia management. Descriptive statistics were applied to summarise the data. Measures such as mean, median, mode and standard deviation (s.d.) were calculated for individual items to provide an overview of central tendency and variability within responses. These descriptive measures allowed for the identification of patterns and trends in communication practices, including the most common modes of interaction, the levels of mutual understanding and openness, and the areas where frustration or miscommunication occurred.

### Ethical considerations

Ethical approval and permission for the research conducted were granted by the Health Research Ethics Committee (HREC) at Stellenbosch University (reference number: N19/10/149). The principles of medical ethics (autonomy, beneficence, non-maleficence and justice) were upheld during the data collection process (Varkey, 2021). Informed consent was obtained from all participants by allowing them to opt into completing the survey after they had read the participant information sheet. As surveys were completed anonymously, the confidentiality of participants was maintained. It can be stated with confidence, therefore, that this survey was completed voluntarily, and the participants remained anonymous to the researchers.

## Results

A total of 18 participants met the inclusion criteria and were included in the study. In terms of job titles, 14 (78%) of the 18 participants were registered nurses. The remaining nurses (*n* = 4) worked as staff or as enrolled auxiliary nurses. The 18 participants were employed in a variety of clinical settings, as displayed in Table 1.

**TABLE 1:** Participants’ clinical settings (*N* = 18).

Clinical setting	Public sector†		Private sector‡		Total responses	
	*n*	%	*n*	%	*n*	%
Clinics	2	15	0	0	2	11
Private practice	0	0	1	20	1	6
Acute hospital setting	2	15	2	40	4	22
Rehabilitation (In-patient)	0	0	1	20	1	6
Rehabilitation (Out-patient)	0	0	0	0	0	0
Primary healthcare sector	2	15	0	0	2	11
Secondary healthcare sector	3	23	0	0	3	17
Tertiary healthcare sector	4	31	0	0	4	22
Other: Research	0	0	1	20	1	6

Note: Percentage values were rounded up to the nearest whole number.

† , *n* = 13 (72%);

‡ , *n* = 5 (28%).

Participants in the public sector worked in tertiary healthcare (*n* = 4), secondary healthcare (*n* = 3), primary healthcare (*n* = 2), acute hospital settings (*n* = 2) and clinics (*n* = 2). Participants in the private sector reportedly worked in private practices (*n* = 1), acute hospital settings (*n* = 2), in-patient rehabilitation (*n* = 1) and research (*n* = 1).

Table 2 reveals that most participants (72%) worked in the public health sector, while 28% worked in the private sector. Participants varied in their years of general nursing experience and in their experience of managing feeding and/or swallowing difficulties.

**TABLE 2:** Professional demographics of participants.

Category	Subcategory	*n*	%
Health sector	Public	13	72
	Private	5	28
General work experience	6 months – 5 years	4	22
	6 – 10 years	4	22
	11 – 15 years	5	28
	16 – 20 years	2	11
	> 20 years	3	17
Experience with feeding/swallowing management (years)	0 – 5	5	28
	6 – 10	3	17
	11 – 15	5	28
	16 – 20	2	11
	> 20	3	17

Note: Percentage values were rounded up to the nearest whole number.

Table 2 provides an overview of the professional demographics of the participants, including health sector, years of general nursing experience and experience in managing feeding and/or swallowing difficulties. These characteristics provide context for the subsequent findings on communication practices that are divided into mutual understanding, openness, frustration with interaction and experienced relevance of and satisfaction derived from communication between nursing personnel and SLTs.

### Mutual understanding

Results of this study indicate that 76% (*n* = 13) of the participants engage in discussions with SLTs regarding the management and feedback of patients with feeding and swallowing difficulties, with these conversations typically initiated by the SLTs. The content of these discussions primarily focuses on patients’ signs and symptoms of feeding and/or swallowing difficulties (66%) and signs of aspiration (27%). During these interactions, 61% of the participants (*n* = 11) felt they were able to express their opinions on patient management, while only one participant reported that nursing input was never considered. In addition, 55% (*n* = 10) of the participants noticed that discussions with SLTs were also used to resolve disagreements about patient care. These exchanges contribute to mutual understanding, which depends heavily on the clarity of both verbal and non-verbal communication.

However, not all participant nurses consistently understood SLT instructions. While 11% (*n* = 2) of the participants reported frequently struggling to understand verbal instructions, 17% (*n* = 3) stated they never understood them. Of the remaining participants, 55% (*n* = 10) said they sometimes understood verbal communication and 17% (*n* = 3) reported rarely understanding it. Although some challenges are evident, it is encouraging that the majority of participants found at least occasional clarity in both verbal and non-verbal communication from SLTs. These findings underscore the importance of improving communication strategies to support mutual understanding in multidisciplinary care.

### Communication openness

The openness of communication between nursing personnel and SLTs is an important aspect of effective collaboration. Communication openness refers to how effortlessly individuals share information and achieve mutual understanding. In this study, 56% of the participants (*n* = 10) reported always experiencing open communication with SLTs, while 11% (*n* = 2) said they often experienced this openness. However, between 28% and 39% of the participants (*n* = 5; *n* = 7) reported sometimes struggling with open communication, and one participant indicated they often struggled to communicate openly with SLTs. Listening is a key factor in promoting openness. In all, 33% (*n* = 6) of the participants felt they were always listened to, 22% (*n* = 4) felt listened to often and 44% (*n* = 8) said they were sometimes listened to. Encouragingly, no participants reported feeling rarely or never listened to. In terms of receiving sufficient advice regarding feeding and swallowing, 44% (*n* = 8) reported always receiving adequate information, 50% (*n* = 9) experienced this often or sometimes and only one participant felt they rarely received enough advice.

Openness in communication also includes the ability to concur with instructions and express concerns freely. One way this can be supported is by accommodating each other’s schedules. Half of the participants (*n* = 9) indicated that SLTs and nursing personnel always or often consider each other’s schedules when developing treatment plans, while 44% (*n* = 8) of the participants reported this happens only sometimes, and one participant said it rarely occurs. Comfort in executing SLT instructions also reflects communication effectiveness. The majority of the participants (*n* = 14; 78%) felt comfortable carrying out instructions related to the management of feeding and swallowing difficulties, while 22% (*n* = 4) felt comfortable only some of the time. Notably, no participants reported feeling uncomfortable performing these tasks, suggesting a generally high level of trust and clarity in communication between nursing staff and SLTs.

### Frustration with interaction

Some of the emotions experienced by nursing personnel after interacting with SLTs included frustration, anger, feeling misunderstood and being overwhelmed. Interestingly, half of the participants (*n* = 9) reported never experiencing anger after interacting with SLTs, while the other half either rarely (*n* = 8) or never (*n* = 1) experienced anger. A large majority (89%) never felt frustrated during these interactions, with only 11% (*n* = 2) reporting some level of frustration. Most participants (*n* = 10) indicated they rarely felt misunderstood and 33% (*n* = 6) stated they never felt misunderstood. While 33% (*n* = 6) reported sometimes feeling overwhelmed, 10 participants (56%) noted they rarely felt overwhelmed by the amount of information provided by SLTs related to feeding and/or swallowing issues. These findings suggest that while negative emotions are minimal, they are still present and worth addressing to improve collaboration.

In terms of frequency of interaction, 44% (*n* = 8) reported engaging with SLTs 1–2 times per week, 33% (*n* = 6) said they interact with SLTs daily about patient care, while 11% (*n* = 2) of the participants reported interacting 3–4 times a week. Notably, 11% (*n* = 2) indicated they never interact with SLTs on a weekly basis – presumably, then, interaction only occurs sporadically. Frequency of interaction may influence the quality of communication and the effectiveness of feedback in managing feeding and swallowing difficulties. While 17% (*n* = 3) of the participants felt their feedback was never regarded by SLTs, less than half (*n* = 8) indicated that their feedback was rarely (*n* = 5) or sometimes (*n* = 3) disregarded. Encouragingly, the majority (*n* = 10) reported that their feedback was never dismissed by SLTs during discussions on patient care, highlighting a generally respectful and inclusive communication dynamic.

### Relevance and satisfaction

Half of the participants (*n* = 9) reported always feeling pleased and satisfied after interacting with SLTs, while 56% (*n* = 10) of the participants indicated that they always felt respected. While 33% (*n* = 6) often felt pleased and satisfied, 22% (*n* = 4) often felt respected during interactions with SLTs. On the other hand, 17% (*n* = 3) only sometimes felt pleased and satisfied and 11% (*n* = 2) only sometimes felt respected. Although none of the participants reported rarely or never feeling pleased and satisfied, 11% (*n* = 2) reported rarely feeling respected. Notably, no participants reported never feeling respected. In all, 39% (*n* = 7) reported always seeking advice, 17% (*n* = 3) often seek advice, another 39% (*n* = 7) sometimes seek advice and one participant rarely seeks advice. None of the participants reported never seeking advice. This information highlights the perceived importance and relevance of SLTs’ input among nursing personnel.

When asked about the relevance of information provided by SLTs, half of the participants (*n* = 9) stated that it was always relevant, 33% (*n* = 6) felt it was often relevant and 17% (*n* = 3) indicated it was sometimes relevant. Furthermore, when questioned about compliance with instructions regarding feeding and/or swallowing problems, 56% (*n* = 10) reported always following SLT instructions, 33% (*n* = 6) followed instructions sometimes and 11% (*n* = 2) followed them occasionally. As communication can occur through various modes, and individual preferences in this regard differ, understanding preferred communication methods is crucial for achieving effective management of patients with feeding and/or swallowing difficulties.

Table 3 presents the descriptive statistics for the four key dimensions of communication between nursing personnel and SLTs: mutual understanding, openness, frustration with interaction and relevance and satisfaction. Table 3 presents descriptive statistics derived from participant responses to the questionnaire. Each dimension was measured using a 5-point Likert scale (1 = never, 5 = always). The mean, median and s.d. reflect the central tendency and variability of participants’ ratings.

**TABLE 3:** Mean, median and standard deviation for dimensions of communication practices.

Communication dimension	Mean	Median	s.d.
Mutual understanding	3.85	4.5	1.07
Openness	3.91	4.0	0.86
Frustration with interaction	1.72	2.0	0.72
Relevance and satisfaction	3.94	4.0	0.92

s.d., standard deviation.

The results show that nursing personnel generally perceive communication with SLTs positively across the four key dimensions. For *Mutual Understanding*, the mean score of 3.85 and median of 4.5 on the 5-point scale suggest that most participants felt they understood SLT instructions and were understood themselves. However, the higher s.d. of 1.07 indicates that these perceptions were not uniform, with some participants reporting weaker experiences of mutual understanding. *Openness* had a mean of 3.91 and a median of 4.0, with a relatively small s.d. of 0.86, showing that participants commonly felt able to share information and be listened to, with fairly consistent agreement across the group. In contrast, *Frustration with Interaction* scored low (mean = 1.72, median = 2.0), and the small s.d. (0.72) suggests that participants consistently reported little frustration, such as anger, feeling misunderstood or being overwhelmed. *Relevance and Satisfaction* received the highest overall ratings (mean = 3.94, median = 4.0, s.d. = 0.92), showing that most participants regarded their interactions as meaningful, respectful and satisfactory, although again with some variation. Taken together, the findings suggest that nursing personnel experience communication with SLTs as largely effective, with low frustration and high satisfaction, but with some room to improve consistency in mutual understanding.

### Modes of communication

The survey explored nursing personnel’s preferred communication methods with SLTs across various clinical interactions. Participants selected their top three communication modes, written, face-to-face verbal, email, instant messaging and question-answer platforms, for different management activities. Notably, no participants chose electronic platforms (e.g., SharePoint) or video conferencing (e.g., Teams), and thus these were excluded from the analysis. For immediate nursing care, 78% of the participants selected written communication as their first preference. Second and third preferences primarily favoured face-to-face verbal and instant messaging. Similarly, when receiving instructions on feeding and/or swallowing management, 83% of the participants preferred written communication as their first choice, followed by face-to-face verbal and instant messaging for second and third preferences, respectively. Feedback to SLTs and supportive activities such as document orders and referral sharing showed a consistent preference pattern, with face-to-face written and verbal communication dominating, while instant messaging appeared mainly as a third-choice option.

Majority of the participants (89%, *n* = 16) also commented on their preferences regarding the management of feeding and/or swallowing difficulties. Instructions that are brief and to the point were preferred by 31% (*n* = 5) of the participants. One participant indicated a preference for an in-depth discussion when receiving instructions about feeding and/or swallowing difficulties. Furthermore, 63% (*n* = 10) of the participants indicated preferences that were covered in the survey, such as face-to-face written and verbal communication or instant messaging.

Open-ended responses offered further insight. Open-ended questions were asked that focus on how nursing personnel would like to receive instructions and information regarding feeding and/or swallowing disorders. Although response rates were low, possibly because of unfamiliar terminology, time constraints or device limitations, 89% (*n* = 16) of the participants shared preferences regarding feeding and/or swallowing management. Most favoured brief, concise instructions, with one participant preferring in-depth discussions. In addition, 44% (*n* = 8) of the participants provided additional reflections on communication practices. While two emphasised WhatsApp as a preferred method, others highlighted generational differences or praised SLT interactions for improving patient risk management. Overall, communication preferences were shaped by context, experience and openness during interactions. The exclusion of electronic forms and video conferencing as preferred modes suggest a continued reliance on direct, personal communication strategies such as face-to-face exchanges and instant messaging.

The survey allowed participants to add additional comments and thoughts. Additional comments about communication practices in exchanges between nursing personnel and SLTs were shared by 44% (*n* = 8) of the participants. One participant made a point of noting that most of the younger generation of nursing personnel do not interact with or are not interested in interacting with SLTs. Two participants indicated that WhatsApp is one of their preferred modes of communication and the remaining participants (*n* = 5) stated that they value their interactions with the SLTs because it is a good way to manage risks such as aspiration.

The findings indicate that nursing personnel and SLTs interact across diverse clinical contexts and that communication practices can be influenced by sector, years of experience and preferred modes of communication. While mutual understanding and openness were generally evident, challenges related to clarity of instructions and occasional frustration emerged. Preferences for written and face-to-face communication underscore the importance of accessible, direct strategies in supporting effective collaboration, while limited inclusion of electronic platforms as communication method of choice highlights potential barriers to digital communication. Taken together, these findings point to both strengths and gaps in interprofessional collaboration.

## Discussion

This discussion interprets the findings of the study in relation to existing literature, in accordance with the five key dimensions of communication between nursing personnel and SLTs.

The first dimension, mutual understanding, emerged as a fundamental aspect of effective interdisciplinary communication. Mutual understanding is often negatively influenced by the SLT’s use of dysphagia-specific terminology and unfamiliar management techniques. Instructions concerning posture adjustments or dietary modifications, for example, may be misunderstood or not fully implemented because of limited training or clinical exposure, which would lead to unfamiliarity with both terminology and techniques (Robbertse & De Beer, 2020). Furthermore, this gap is often exacerbated when SLTs are not consistently present in the ward environment, making it difficult for nursing personnel to seek clarification or engage in follow-up discussions. Authors such as Blackwell and Littlejohns (2010) and Pascoe and Norman (2011) have long cautioned that working in professional silos, a lack of cultural sensitivity and infrequent interactions between team members can affect mutual understanding, which in turn influences the collaborative efforts in patient care. In accord with our findings, a recent systematic review by Armstrong et al. (2023) reveals that shared knowledge, role clarity and respect between professionals are crucial for effective interprofessional collaboration. Encouragingly, most participants in this study felt that SLTs acknowledged their opinions and valued their practical experience. This suggests a foundation of respect upon which stronger collaborative relationships and future communication could be built.

Mutual understanding cannot be based solely on respect; it requires frequent engagement, role clarity and feedback. Nursing personnel, particularly those with less experience or training in dysphagia management, may require more detailed and patient instruction from SLTs. In line with the arguments put forward by Kuokkanen and Leino-Kilpi (2000), empowering nursing personnel with knowledge and involving them in decision-making can enhance confidence, ownership, and ultimately, mutual understanding. Integrating regular interdisciplinary discussions with specific handover strategies can assist with effective communication and interaction regarding patient care.

The second dimension, communication openness, relies on mutual understanding. While some participants expressed that they felt heard and respected in interactions with SLTs, others reported feeling overlooked or dismissed. These mixed experiences highlight the variability of communication openness across context and individuals. Openness in communication implies a bidirectional exchange where questions, concerns and suggestions are embraced without judgement. However, several contextual barriers negatively influence the practice of communication openness, for example, staff shortages and time constraints. Nursing personnel often reported that the intermittent availability of SLTs in the ward does not align with the unpredictability of acute and rehabilitation care, and limits opportunities for spontaneous case discussions and interactions (Robbertse & De Beer, 2020). In line with our findings, Templeton (2025) reports that interprofessional simulations (a healthcare education method where students from different professions work together in a simulated environment to learn and practice collaborative teamwork and communication) between nursing and SLT students foster mutual respect and enhance communication openness by creating safe spaces for dialogue and collaborative problem-solving. These findings align with those of Wieke Noviyanti et al. (2021), emphasising that interprofessional training in communication strategies can cultivate a culture of openness and inclusivity in clinical practice.

The third (relevance and satisfaction) and fourth (frustration with interactions) dimensions were interconnected. Emotional responses from nursing personnel, which ranged from respect and satisfaction to frustration, revealed how communication experiences can affect interprofessional relationships. While participants generally respected the SLTs and expressed a willingness to cooperate, only half reported feeling satisfied with the interactions. Nursing personnel sometimes question the relevance of SLT instructions, considering their daily routines and patient needs, citing a lack of dysphagia-appropriate training and unclear clinical rationale. This aligns with findings from a recent quasi-experimental study by Hur and Kang (2024), which showed that communication training programmes for nursing personnel caring for patients with communication or swallowing difficulties significantly improved the perceived relevance of and satisfaction with interprofessional interactions. The study reveals how targeted professional development and training can enhance nursing personnel’s engagement and satisfaction. These findings suggest that addressing relevance and satisfaction in practice requires SLTs to provide ongoing professional development while considering the practical constraints of ward environments.

Frustration with interactions often results from both contextual issues and interprofessional dynamics. A lack of feedback or follow-up from SLTs, for example, was shown to have left some nursing personnel uncertain about whether they had implemented care plans adequately. Nursing personnel may also feel overwhelmed by quick verbal instructions or documentation based on dysphagia terminology that is poorly understood, particularly when they are faced with patients who are at high risk for aspiration or malnutrition. Similar findings have been reported by Wieke Noviyanti et al. (2021), who emphasised that inadequate communication practices in hospital settings can lead to frustration among nurses and negatively impact patient safety culture. Deliberate communication practices need to be adopted by SLTs, focusing on repetition of key points, checking for understanding and ensuring that written instructions are unambiguous.

The fifth dimension, modes of communication, revealed important insights into how information is shared and received in clinical settings. Face-to-face and written communication were most commonly used and preferred. These modes were perceived as reliable, familiar and conducive to clarifying instructions or asking questions. Verbal and written communication seem to remain the most effective and trusted modes among South African healthcare professionals. In contrast, electronic communication, such as teleconferencing apps or emails, was less frequently used by participants in the current study. Barriers included limited access to digital devices, a lack of formal training in digital communication tools and infrastructural challenges such as inconsistent electricity and poor internet connectivity (Abbott & Coenen, 2008; Goldberg, 2015). Although platforms such as WhatsApp offer encrypted, instantaneous messaging (De Benedictis et al., 2019), concerns about professionalism, medico-legal accountability and the permanence of communication persist. Integrating electronic communication into professional practice would require clear institutional guidelines, access to devices and ongoing support. The importance of using multimodal communication strategies, combining verbal, written and visual tools, to enhance clarity and ensure consistency in patient management (Hess, 2016; Pain et al., 2016) does not necessarily imply the use of advanced technology. In busy ward environments, quick-reference guides, visual checklists and simplified communication charts could support effective information transfer.

A final consideration is the influence of generational and experiential differences among nursing personnel. Senior nurses, who may have developed routines over many years, sometimes rely on different vocabulary and approaches than younger staff do. Generational diversity can affect communication preferences, with younger professionals more inclined to embrace digital communication, while older staff may prioritise traditional, face-to-face interactions (Daly, 2017). As also reported by Kınas et al. (2025), experience strongly influences confidence and competence in clinical communication; nurses with more years of practice tend to feel more comfortable interacting with SLTs and are more likely to implement recommendations accurately. Experience also plays a critical role in confidence and competence when managing complex patient needs. Those with more years in the field tended to express greater comfort when interacting with SLTs and were more likely to implement recommendations with fidelity.

In conclusion, improving communication between nursing personnel and SLTs requires attention to all five dimensions explored in this study. Promoting mutual understanding through shared language and regular contact, fostering communication openness by creating inclusive dialogue spaces, enhancing relevance and satisfaction through contextualised training, reducing frustration by standardising communication practices and leveraging appropriate modes of communication can collectively strengthen interdisciplinary collaboration. As a result, patient outcomes in the management of feeding and swallowing disorders can be significantly improved. This underscores the importance of interprofessional education, structured communication systems and institutional commitment to collaborative practice in the South African healthcare context.

### Limitations and future research

This study had some limitations, notably with regard to data collection using e-surveys and sample size. Using e-surveys may exclude nursing personnel with limited access to digital devices or knowledge of completing e-surveys, which introduces possible selection bias. The small sample size and reliance on convenience sampling further limit the representativeness of the findings and the ability to generalise results to all nursing personnel and SLTs across South African healthcare settings. Specifically, the limited number of enrolled nursing assistants (ENAs) and staff nurses made it impossible to compare communication practices between different nursing groups or evaluate how experience levels influence communication. These methodological constraints may affect the validity of conclusions, and cautious interpretation is therefore advisable.

Future research should consider addressing the limitations of the study by conducting face-to-face surveys and extending data collection periods to reach a larger and more diverse sample. Future studies could explore nursing personnel’s help-seeking behaviours when communication challenges arise, the influence of nursing experience on communication practices and the impact of electronic devices on communication effectiveness. Further research should also investigate communication between SLTs and dieticians in managing feeding and swallowing difficulties, given dieticians’ roles in nutrition optimisation and training others in feeding management. Such research would provide more context-specific evidence to inform training programmes, institutional policies and interprofessional collaboration strategies in South African healthcare settings.

### Clinical implications

This study offers several clinical implications for improving communication between nursing personnel and SLTs in the management of feeding and swallowing difficulties. Effective communication should be clear, concise, complete and timely (Wang et al., 2018). Speech- language therapists need to allocate dedicated time for patient management discussions while considering nursing staff schedules. Increasing regular check-ins can help prevent misunderstandings and provide nursing personnel with opportunities to express their views and resolve disagreements, thus fostering mutual understanding. Structured communication strategies such as SBAR (Situation-Background-Assessment-Recommendation) can promote communication openness through formalised discussions where nursing personnel can express concerns and ask questions within a safe environment.

In addition, SLTs should acknowledge the critical role that nurses play in feeding and swallowing management and expand educational opportunities through in-service training and external workshops that focus on simulation exercises and demonstration of feeding techniques. Providing educational opportunities that are relevant to the context and realities of the ward could enhance satisfaction with interdisciplinary communication. Frustration can be addressed through structured feedback mechanisms that allow anonymous feedback to SLTs. Incorporating checklists and follow-up tools that are agreed upon by the interdisciplinary team can possibly promote confidence, but also accountability.

Regarding modes of communication, while written communication is the preferred mode, it should be complemented by verbal communication. In the same way, spoken communication could be complemented with brief written notes. The integration of electronic communication is encouraged because of its efficiency; therefore, training on electronic devices and relevant programmes should be provided to all staff. When electronic communication is not feasible, alternative preferred methods should be mutually discussed and implemented at the institutional level to ensure effective collaboration. Taking into account both generational and experiential differences, tailored communication strategies are imperative to accommodate preferences and levels and clinical experience. Mentorship, by pairing senior nursing staff with younger staff, may bridge the communication gap but also ensure knowledge transfer and mutual learning.

## Conclusion

Mutual understanding and effective communication foster respect and consensus between nursing personnel and SLTs, thus building a firm foundation of communication openness. While some mutual understanding currently exists, improvements are necessary. This study showed that nursing personnel may not always fully comprehend SLTs’ instructions, which can affect task execution and adherence. Mutual understanding and communication openness significantly influence the dynamics between nursing personnel and SLTs. Reduced mutual understanding and openness can lead to frustration and dissatisfaction, impacting the perceived relevance of and satisfaction with interactions.

The choice of communication mode is vital in fostering mutual understanding and openness, as it affects how information is received and interpreted. Understanding the availability, accessibility and quality of different communication modes within institutions supports effective interdisciplinary communication. Establishing effective communication practices at the institutional level is critical, aiming for efficiency without compromising patient care. Promoting comprehensive communication practices benefits both staff and patients, and facilitates overall better care outcomes.

The study contributes to the limited research landscape by providing a detailed, multidimensional analysis of SLT-nurse communication, specifically in the field of feeding and swallowing management. The study highlights the communication practices found in exchanges between the SLTs and nursing personnel in low- and middle-income countries, such as South Africa, in light of contextual and resource constraints. This study provides empirical evidence regarding how communication occurs, the challenges faced and the factors influencing effectiveness in clinical practice. The findings inform targeted strategies for clinical training, institutional policy and interdisciplinary collaboration, offering evidence-based recommendations to enhance communication, reduce misunderstandings and improve patient care outcomes. By integrating these contributions, this study contributes both theoretical and practical value to improve communication between SLTs and nursing personnel in clinical settings.
